# The Microbiota and the Gut–Brain Axis in Controlling Food Intake and Energy Homeostasis

**DOI:** 10.3390/ijms22115830

**Published:** 2021-05-29

**Authors:** Marina Romaní-Pérez, Clara Bullich-Vilarrubias, Inmaculada López-Almela, Rebeca Liébana-García, Marta Olivares, Yolanda Sanz

**Affiliations:** Microbial Ecology, Nutrition & Health Research Unit, Institute of Agrochemistry and Food Technology, National Research Council (IATA-CSIC), 46980 Valencia, Spain; marina.romani@iata.csic.es (M.R.-P.); clarabullich@iata.csic.es (C.B.-V.); inma.lopez@iata.csic.es (I.L.-A.); reliegar@iata.csic.es (R.L.-G.); m.olivares@iata.csic.es (M.O.)

**Keywords:** microbiota, gut–brain axis, nutrient sensing, food intake and obesity

## Abstract

Obesity currently represents a major societal and health challenge worldwide. Its prevalence has reached epidemic proportions and trends continue to rise, reflecting the need for more effective preventive measures. Hypothalamic circuits that control energy homeostasis in response to food intake are interesting targets for body-weight management, for example, through interventions that reinforce the gut-to-brain nutrient signalling, whose malfunction contributes to obesity. Gut microbiota–diet interactions might interfere in nutrient sensing and signalling from the gut to the brain, where the information is processed to control energy homeostasis. This gut microbiota–brain crosstalk is mediated by metabolites, mainly short chain fatty acids, secondary bile acids or amino acids-derived metabolites and subcellular bacterial components. These activate gut–endocrine and/or neural-mediated pathways or pass to systemic circulation and then reach the brain. Feeding time and dietary composition are the main drivers of the gut microbiota structure and function. Therefore, aberrant feeding patterns or unhealthy diets might alter gut microbiota–diet interactions and modify nutrient availability and/or microbial ligands transmitting information from the gut to the brain in response to food intake, thus impairing energy homeostasis. Herein, we update the scientific evidence supporting that gut microbiota is a source of novel dietary and non-dietary biological products that may beneficially regulate gut-to-brain communication and, thus, improve metabolic health. Additionally, we evaluate how the feeding time and dietary composition modulate the gut microbiota and, thereby, the intraluminal availability of these biological products with potential effects on energy homeostasis. The review also identifies knowledge gaps and the advances required to clinically apply microbiome-based strategies to improve the gut–brain axis function and, thus, combat obesity.

## 1. Gut and Brain Control of Energy Homeostasis

The gastrointestinal tract is a huge sensory organ able to transmit nutrient-related information to the brain where diverse endocrine and neural inputs converge to ultimately control feeding behaviour and whole-body energy homeostasis through efferent outputs. Nutrient sensors are receptors that bind molecules derived from the macronutrient digestion. They are located in enteroendocrine cells (EECs), which are the primary chemosensory cells in the gut, and are in direct contact with the luminal environment [1]. The activation of nutrient sensors of EECs initiates the secretion of gut hormones that in turn trigger the downstream processes required to maintain energy homeostasis postprandially. The most extensively studied gut hormones are cholecystokinin (CCK), gastric inhibitory polypeptide (GIP), mainly secreted in the upper part of the intestine, and glucagon-like peptide-1 (GLP-1) and peptide tyrosine tyrosine (PYY), mainly secreted in the distal part [2]. EECs specifically sense carbohydrates, proteins and lipids through a diverse repertoire of nutrient sensors. Among others, sodium/glucose cotransporter 1 (SGTL1) mediates carbohydrate sensing, mainly sensed in the form of glucose, and, then, induces GIP and GLP-1 secretion; calcium-sensing receptor (CaSR) senses dietary amino acids and, then, secretes CCK and GLP-1; and G-protein coupled receptor (GPR) 120 (FFAR4), GPR40 (FFAR1) and GPR119 sense products from lipid digestion and induce the secretion of CCK, GIP, GLP-1 and PYY [1,3]. EECs are in close proximity to vagal afferent nerve terminals in the lamina propria, which express intestinal hormone receptors. Enteroendocrine hormones secreted after a meal activate nutrient-sensing signalling via endocrine routes, when gut hormones reach the brain or other organs through systemic circulation, or via paracrine routes, when hormones stimulate vagal afferents nearby in the intestinal mucosa and, then, the signal reaches the brain.

The arcuate nucleus (ARC) of the hypothalamus is accessible to humoral signals since is not fully protected by the blood–brain barrier [4]. The ARC contains two subpopulations of neurons; those expressing anorexigenic propiomelanocortin (POMC), the precursor of α-melanocyte-stimulating hormone (αMSH), and the cocaine and amphetamine regulated transcript (CART); and those neurons expressing the agouti gene-related peptide (AgRP) and neuropeptide Y (NPY) [5]. The activation of the POMC/CART neurons after feeding induces the release of α-melanocyte stimulating hormone (α-MSH) that binds to melanocortin 4 receptor (MC4R) in the paraventricular nucleus (PVN) where the information is integrated to suppress food intake and regulate body weight [6]. Thus, the increased levels of gut hormones such as CCK, GLP1 or PYY after a meal reach, via endocrine routes, the ARC to suppress food intake [7]. Via paracrine routes, gut hormones can stimulate vagal afferents [8], which are bipolar neurons whose somas converge in the nodose ganglion and their proximal extensions terminate in the nucleus of the solitary tract (NTS) in the brainstem [9]. This, together with the hypothalamus, represents an important integrative gut–brain hub. The NTS also contains POMC neurons and, through monoamine neurotransmission, transmits sensory information to upstream brain areas including the PVN and the dorsal vagal complex (DVC) that send efferent outputs involved in the vago–vagal reflex [5]. In summary, there are different paths for transmitting nutrient signals to the brain. The hypothalamus senses nutrients through the action of enteroendocrine hormones released in the intestine, which reach the brain by humoral pathways [10,11,12], that is through the circulatory system, or by paracrine pathways, that is by activating the nerve terminals of intestinal vagal afferents, which are the main focus of the present review.

The increased intake of energy-dense and palatable foods impairs the brain circuits controlling energy homeostasis, whose deficient response to nutrient signals alters feeding behaviour, which contributes to obesity. Accordingly, the restoration of nutrient signalling via the gut–brain axis represents a promising strategy to improve the central control of energy homeostasis in response to meals and, thus, help combat obesity [13,14,15]. The gut microbiota is a biological factor that might directly or indirectly influence nutrient-sensing and, theoretically, its modulation could aid in the restoration of gut-to-brain communication and maintaining energy homeostasis. The role of gut microbiota in obesity has been proven through faecal transplantation experiments, which produce the metabolic phenotype of the donor in the recipient organism [16]; however, the mechanisms by which microbiota influence energy homeostasis and body-weight regulation are not yet fully understood. Western diets, rich in saturated fat and simple sugars, alter the gut ecosystem reducing bacterial diversity and increasing the abundance of potential pathogens [17]. This, in turn, could alter the metabolism of macronutrients and, thereby, the ligands available for nutrient sensors in the intestinal lumen as well as the presence of structural microbial components that might also act as ligands of sensors that mediate the gut-to-brain communication. Sensors of microbially produced metabolites and bacterial components are located in EECs, vagal afferents and, occasionally, in the hypothalamus and can be activated by ligands reaching the brain through the systemic circulation. These receptors sense microbial-derived metabolites such as short chain fatty acids (SCFAs), secondary bile acids (BAs) and amino acid-derived metabolites and subcellular bacterial components such as caseinolytic peptidase B (ClpB), lipopolysaccharide (LPS) or muramyldipeptide (MDP). 

Here, we review the role of dietary factors, including feeding patterns and diet composition, in modulating gut microbiota structure and function and potentially affecting gut-to-brain nutrient sensing, mainly via endocrine and neural routes. We also compile evidence of microbial molecules able to modulate specifically hypothalamic-mediated control of the energy homeostasis, placing special emphasis on their role in regulating food intake and, thereby, obesity. 

## 2. Circadian Rhythms, Eating Patterns and Gut Microbiota in Energy Homeostasis Control

Most of the physiological functions display circadian rhythms. At cellular level, these are governed by clock proteins that generate self-sustained daily oscillations of the biological processes that allow cells to anticipate and thus to optimally respond and adapt to environmental variations [18,19]. In mammals, most cells throughout the body express clock genes that elicit autonomous circadian oscillations [20]. The hypothalamic clock located in the suprachiasmatic nucleus acts as a master pacemaker that synchronizes the secondary clocks [21,22]. Eating behaviour and energy metabolism show a well-defined circadian pattern through the day. In humans and rodents, the timed feeding patterns are governed by metabolic hormones and nutrients [23] and shift certain secondary clocks without affecting the suprachiasmatic nucleus, whose circadian oscillations are mainly governed by light–dark cycles [24,25]. Circadian rhythmicity, specially that affecting eating behaviour, is required to maintain energy homeostasis. Indeed, humans with disrupted circadian rhythms have higher risk of developing obesity and type 2 diabetes (T2D) [26,27] and mistimed eating increases adiposity in rodents [28] and also favours obesity and increases postprandial glycaemia in humans [29,30].

Circadian rhythmicity is involved in gut microbiota–host interactions. Gut microbiota play a role in maintaining the circadian rhythms of the host, including those related to eating patterns and energy homeostasis, and vice versa. The abundance of bacterial species, as well as of their derived metabolites, vary throughout the day, suggesting associations between gut microbes and eating behaviours in humans [31]. A recent study by Reitmeier et al. [32] supports the link between diurnal fluctuations of the gut microbiota and metabolic health in humans. The authors identified these diurnal oscillations in faecal samples of three independent large-scale population studies and detected aberrant oscillations in subjects with metabolic disorders such as obesity or T2D [32]. 

### 2.1. Gut Microbiota Influences Circadian Rhythms Affecting Metabolism 

The influence of gut microbiota on the host circadian rhythms and on energy metabolism has been demonstrated in peripheral tissues such as the gut, liver or white adipose tissue. In mice, gut microbiota is required to maintain the circadian rhythmicity of the expression of the Toll-like receptors (TLRs), through which the microbiota-associated molecular patterns (MAMPs) communicate with the intestinal epithelial cells (IECs) of the host [33]. The absence of gut microbiota impairs the circadian clock of the IEC leading to an increased synthesis of ileal corticosterone, which impairs systemic metabolic homeostasis. This is evidence for the role of the microbiota–IEC dialogue oscillation during the day in metabolic health maintenance [33]. Similarly, germ free mice show altered expression of circadian-related genes in liver and white adipose tissue [34]. Microbiota-derived molecules, especially those that activate aryl hydrocarbon receptor (AHR) and pregnane X receptor (PXR), are crucial to maintain the rhythmicity and sexual dimorphism of the growth hormone secretion, which maintains rhythmic gene expression and metabolome in liver in a sex-dependent manner [34].

### 2.2. Eating Patterns, Gut Microbial Diurnal Oscillations and Energy Homeostasis

The eating rhythms of the host cause microbiota oscillations, which seem flexible and able to adapt to the nutritional environment. Mice deficient in the clock gene *Per1/2* or mice submitted to an experimental “jet-lag” show altered eating rhythms throughout the day linked to aberrant microbiota fluctuations [35]. Time-restricted feeding of *Per1/2* knock-out mice partially restores the microbiota oscillations, proving that eating behaviour is the main driver of microbiota diurnal oscillations [35]. A fat-rich diet also blunts the cyclical changes of the gut microbiota [36,37] and, consequently, their derived-metabolites, thus affecting host metabolism. For instance, the fluctuations of species belonging to the family Lachnospiraceae and their derived metabolite butyrate disappear under a high fat diet (HFD), which affects hepatic clock genes and metabolism [36]. Additionally, the spatial distribution of microbes in the gut seems to follow daily fluctuations. In rodents, bacterial adherence to the intestinal epithelium and their proximity to the mucosal surface are higher in the dark than in the light phase and, over the course of the day, some bacterial species show oscillations of epithelial adherence, findings that are dependent on feeding time [38]. Importantly, the nutrient-induced microbiota oscillations throughout the day lead to fluctuations of luminal and serum metabolites, such as amino acids and polyamines, responsible for the circadian hepatic transcriptome required for physiological processes such as detoxification functions [38]. Eating rhythmicity persists in antibiotic-induced microbiota depletion [38] but obese-associated hyperphagia might be transmissible through gut microbiota transplants to germ-free mice; this occurs in hyperphagic TLR5 knock-out mice [39] although not in *ob*/*ob* mice. This suggests that eating rhythmicity is driven mainly by the host circadian clock machinery driving endocrine hormone secretion, but nutrient signalling could also be modulated by gut microbiota activated pathways. 

Interestingly, the diurnal bacterial growth dynamics, which are controlled by bacterial quorum sensing, availability of nutrients, gut motility and immunity, apparently overlap with host-feeding cycles [40]. In this regard, a bacterial growth-based model of appetite control has been proposed, in which the exponential and stationary growth phases of the bacteria are associated with satiety-induced signals of the prandial and postprandial phase, respectively, while the decline growth phase is coupled with hunger-related signals, defining intermeal intervals [40]. Therefore, to understand how the gut microbiota shapes eating rhythms it is necessary to investigate the bacterial growth fluctuations parallel to the generation of dietary and bacterially derived ligands of nutrient sensors able to centrally control energy homeostasis (such as SCFAs, secondary BAs or amino acid-derived metabolites—see Section 4 for details). For instance, ClpB, an antigen mimetic of αMSH produced by *Escherichia coli* [41], activates host satiety pathways following nutrient-induced bacterial growth [42]. Similarly, nutrient-induced bacterial growth might enhance the generation and release of bacterial ligands, such as cell wall and membrane components (e.g., lipopolysaccharide, LPS, or muramyldipeptide, MDP) or quorum sensing molecules, which in turn activate nutrient sensing pathways in the gut. Although the causal link between the diurnal oscillations of these molecules and feeding rhythms has yet to be proven, LPS and MDP are known to suppress food intake through immune sensing pathways linked to satiety and sickness behaviour [43,44,45]. Through an immune-related cascade in which LPS and MDP bind to TLR4 and NOD2, respectively, both molecules seem to modulate GLP-1-mediated signalling [46,47,48]. Nevertheless, the extent to which nutrient-induced bacterial growth and the consequent release of LPS or MDP synchronize meal-related host rhythms under physiological conditions is still unknown. Indeed, the effects of LPS and MDP on feeding behaviour have mainly been described in the context of bacterial infection, where these bacterial molecules reach the systemic circulation. However, their role as modulators of nutrient signalling in the gut, for instance as GLP-1 secretagogues, remains mostly unexplored. In this regard, LPS could act as an inducer of GLP-1 secretion when administered orally only if the gut barrier integrity is impaired [46]

## 3. Diet Composition Influences Gut Microbiota and Gut-to-Brain Nutrient-Sensing

Adherence to a particular dietary pattern, such as a Western, vegetarian or Mediterranean diet, or dietary interventions characterized by large variations in the macronutrients proportions, such as high protein or high fat (ketogenic) diets, impacts gut microbiota composition and function [49,50,51]. The composition in macronutrients of these diets exerts an important effect on the availability of microbially derived ligands of dietary and non-dietary nature in the luminal content able trigger gut-to-brain sensing routes and controlling food intake and energy metabolism (summarized in Table 1). The availability of these ligands depends on multiple biological processes, including the microbiota-mediated catabolism of ingested nutrients and their absorption by enterocytes. 

Most of the simple carbohydrates, proteins and fats are absorbed in the proximal regions of the small intestine while indigestible complex carbohydrates, the preferred carbon source of gut microbiota, reach the colon, favouring the growth of anaerobic bacteria and species diversity. The gut microbiota structure and composition are flexible, showing rapid adaptations to macronutrient shifts (24–48 h) that remain for short periods while more permanent changes might require longer adherence to a dietary pattern [52,53]. When the dietary macronutrient composition is high in non- or low-fermentable nutrients (e.g., lipids or proteins) these macronutrients overwhelm their intestinal absorption and reach the colon, where they are used by the best adapted microbes with the subsequent shift in their ability to activate gut nutrient sensing routes. 

Although the diet per se modulates the energy metabolism, here we focus on effects possibly mediated by the interactions between the gut microbiota and the main macronutrients of different diets for which there is substantial scientific evidence. A special emphasis is placed on those interactions that potentially affect gut-to-brain nutrient sensing and, thereby, energy homeostasis.

### 3.1. Western Diets

Over the last two decades, the dietary patterns of modern societies have exhibited a shift towards a diet low in fibre and high in saturated fats, simple sugars and refined foods, termed the “Western diet” [54]. The Western diet is frequently associated with altered eating patterns, leading to hyperphagia and obesity onset. Specifically, the overconsumption of saturated fats and simple sugars in Western diets contributes to impair eating behaviour as a consequence of an overstimulation of the nutrient sensing routes that disturbs how brain senses these nutrient-related signals to control food intake patterns. Particularly, the intake of dietary fats amplifies meal sizes in humans; this phenomenon is exacerbated in obese subjects [55] that also showed reduced PYY secretion following a lipid load [56,57]. Studies in obese individuals reported increased postprandial levels of GLP-1 and CCK and occasional reductions of GLP-1 secretion [58,59] while investigations in rodents demonstrated that HFD diminished the sensitivity of GLP-1 and CCK contributing to alter eating behaviour and energy homeostasis [60,61]. In addition to the effects on eating behaviour, mouse studies also demonstrate that diets rich in saturated fats impair the enteric detection of glucose required to induce glycogen depots in muscle through a GLP-1 receptor mechanism in the arcuate NPY-expressing neurons [62].

Sugar-enriched foods also contribute to increasing weight gain as demonstrated by the meta-analyses of randomised controlled intervention trials and observational studies [63]. Excessive intake of sugar is associated with overeating due to impaired hedonic and homeostatic brain circuits [64,65,66] related to defective gut-to brain glucose sensing. In lean humans, calories from dietary sugars negatively correlate with glucose-induced GLP-1 secretion and positively with dorsal striatal food cue reactivity to palatable food [67]. Obesity in combination with increased dietary sugars has an additive effect reducing circulating levels of GLP-1 after a glucose load [68]. Functional magnetic resonance imaging also reveals that, compared with lean individuals, obese subjects show altered sucrose-related functional connectivity of lateral hypothalamus and NTS with reward-related brain areas resulting in reduced sucrose-associated hedonic responses [69] while studies in mice indicate that the Western diet impairs glucose sensing in POMC-expression neurons [70].

Together with the altered feeding behaviour and energy homeostasis, the Western diet also decreases the bacterial diversity in the human gut, partly due to its reduced content of complex carbohydrates [71] and especially if diets are rich in high saturated fatty acids (SFAs) [72]. 

Compared with children from rural areas of Africa, European children eating a typical Western diet showed a higher abundance of Firmicutes with an overrepresentation of *Faecalibacterium*, while genera from Bacteroidetes, such as *Alistipes* and *Prevotella*, are increased and decreased, respectively [50]. These associations can reflect a metabolic adaptation of the intestinal bacteria to a new nutritional environment. How gut microbiota, through their interactions with Western diet-derived nutrients (i.e., saturated fats and simple sugars), influences the pool of bacterial-derived metabolites potentially activating gut nutrient sensing routes to control eating patterns and energy homeostasis is in an early stage of investigation. Herein, we compile associative studies in humans and mechanistic studies in rodents which provide evidence of these interactions affecting the central control of energy homeostasis. 

In humans, short-term adaptations of the gut microbiota to an animal-based diet consist of a predominance of bile-tolerant microorganisms such as *Alistipes* or *Bilophila* and a decrease in bacteria adapted to metabolize dietary plant polysaccharide such *as Roseburia*, *Eubacterium rectale* and *Ruminococcus bromii* [53]. In vitro human gut simulator assays have demonstrated the predominance of lipid degradation-related genes and the downregulation of carbohydrate degradation genes, along with an increase in Gammaproteobacteria and the genera *Alistipes* and *Bilophila* in bacterial communities exposed to a fat-based medium [73]. In mice, a SFAs-enriched diet, especially high in palmitic acid, boosts overgrowth of *Bilophila wadsworthia*, which aggravates the HFD-induced metabolic disturbances [74]. The increased abundance of *B. wadsworthia* seems to be a secondary consequence of the SFAs on BAs metabolism [75]. Specifically, the SFAs-enriched diet increases the host production of BAs conjugated with taurine, which increase the fitness and growth of *B. wadsworthia*, while inhibiting other bacteria. *B. wadsworthia* also exacerbates the HFD-induced decrease in deoxycholic acid (DCA) and hyodeoxycholic acid (HDCA), secondary BAs that mediate gut nutrient sensing routes [75] (see Section 4 for details).

Studies in mice fed saturated fat-enriched diets combined with microbiota transplants or antibiotic treatments have provided insights into the causal relationship between specific bacterial genera or species and nutrient sensing routes involved in the central control of glucose homeostasis. For instance, the abundance of *Lactobacillus gasseri* (which is reduced by Western diets) helps to sense oleic and linoleic acid in the small intestine to ultimately reduce hepatic glucose production through downregulation of the bile acids receptor farnesoid acid receptor (FXR) and upregulation of the long-chain acyl-CoA synthetase-3 (ACSL3) [76]. In addition, the Western diet-induced lactobacilli decrease seems to be involved in the defective GLP-1 receptor/nitric oxide synthase (nNOS)-mediated signalling in response to oral glucose load to centrally control insulin secretion and gastric emptying, a process initiated by LPS and MDP [47].

Western diet also provides an excess of non-absorbable glucose and fructose to the small intestine and subsequently reach the colon. Gavage of [13C]fructose in mice reveals that high doses of fructose saturate the fructose-to-glucose conversion in the small intestine, enhancing fructose utilization by the microbiota in the colon via the hexokinase pathway to further generate tricarboxylic acid cycle (TCA) intermediates, such as essential amino acids (valine and leucine) and SCFAs (succinate, butyrate and acetate) [77,78]. In addition, sucrose-derived glucose and fructose in the colon downregulate a gene involved in gut bacteria colonization, reducing the fitness of bacterial species such as *Bacteroides thetaiotaomicron* associated with a lean phenotype by some studies [79]. The Western diet-induced decrease in the abundance of *Lactobacillus* spp. in the small intestine also impairs the glucose transporter SGLT1 and the GLP-1 receptor-mediated glucose sensing in the gut required to centrally control energy homeostasis in rats [80].

### 3.2. Vegetarian Diets

Vegetarian diets largely vary in composition according to the interindividual choice of foods. Commonly, they are devoid of meat and can include eggs and dairy products (lacto-ovo-vegetarian, lacto-vegetarian or ovo-vegetarian); exclude eggs and dairy products (vegan diets) or only include vegetables, fruit, nuts, seeds, legumes and sprouted grains (raw vegan diet) [81].

Compared with omnivorous diets, individuals adhering to vegan or vegetarian diets show less uncontrolled eating and emotional eating [82]. In the short-term, compared with a processed meat meal, a plant-based tofu meal enhances the secretion of GLP-1 in T2D individuals with a concomitant increase in satiety and a reduction of triglycerides in plasma [83,84].

Deciphering which are the vegetarian/vegan diet-associated nutrients and molecules that facilitate the gut-to brain nutrient sensing would provide valuable knowledge for designing effective anti-obesity dietary interventions for subjects who chose this dietary pattern. Overall, well-implemented vegetarian diets meet or exceed the recommended fibre intake [81,85]. A comparative study demonstrated that while the daily intake of sugars does not differ between vegan, vegetarian and omnivorous diets and the intake of proteins shows slight differences, subjects who adhered to a vegetarian/vegan diet consumed fewer calories and saturated and monounsaturated fats and higher amounts of dietary fibre [86], pointing out a main role of fibres in the ability of plant-based diets to modulate the gut–brain axis.

Dietary fibres, complex carbohydrates unable to be digested and absorbed in the upper part of the human intestine and which pass to the distal part, serve as substrate for intestinal microbes [87]. These complex polysaccharides, highly abundant in plant-based diets, per se display protective effects against the progression of obesity; moreover, new investigations suggest that the gut microbiota might be an intermediate player of the fibre benefits. Fibre fermentation generates diverse molecules including lactate, pyruvate and succinate as well as the SCFAs including acetate, propionate and butyrate in a molar ratio of 60:20:20, approximately [88]. Specifically, acetate is produced by phosphate acetyltransferase and acetate kinase; propionate is catalysed via the succinate, acrylate and propanediol pathways; and butyrate is produced by phosphate butyryltransferase and butyrate kinase [89].

Overall, an overrepresentation of the genera *Prevotella* and *Ruminococcus* is present in faeces of humans who adhere to plant-based diets [90]. Interventional studies in humans also show that different types of fibres favour the growth of bacteria with the enzymatic machinery directly involved in their fermentation. For instance, the abundance of *Bifidobacterium* spp., whose genome encodes transporters and enzymes involved in complex carbohydrate catabolism [91], are increased in the human colon as a result of dietary supplementation with inulin-type fructans [92], resistant starch [93], galactoologosacharides [94] or arabyno-oligosaccharides, which also increase *Prevotella* [95,96]. Some studies also associate the bifidogenic effect of the inulin-type fructans supplementation with improvements in oral glucose tolerance in obese women and reductions in body weight z-score in obese children [92]. Nonetheless, most of the human studies only show associations between specific bacterial taxa and metabolic benefits, and further studies are needed to demonstrate causality. Thus, it is also possible that the microbiota-mediated metabolic benefits of prebiotic fibres could depend on the initial microbiota configuration of the human subject and its capability to enhance the production of enough fermented products (e.g., SCFAs or others) and that this variation explains the inconsistency of the results across intervention trials [96,97].

Nevertheless, some studies in germ-free mice intentionally colonized with specific microbiotas have already demonstrated that, for example, plant-based diets induce microbiota-dependent benefits. In particular, *Prevotella copri* seems to mediate the fibre-induced glucose tolerance improvement as revealed by a study conducted in germ-free mice colonized with the gut microbiota from individuals that favourably respond to barley kernel-based bread consumption [98]. An important route of communication between the gut and the brain is the intestinal gluconeogenesis that, via portal glucose sensing, is essential for maintaining the postprandial regulation of the hypothalamic energy homeostasis control, including food intake and endogenous production of glucose [99]. It seems that *P. copri* could modulate the gut–brain axis to centrally control glucose homeostasis antagonizing the effects of other intestinal bacteria. Particularly, *P. copri* seems to limit the effects of *Bacteroides thetaiotaomicron* on energy metabolism impairment, possibly due to the ability of the latter bacterium to reduce colonic gluconeogenesis [98]. This idea was later demonstrated by de Vadder et al. 2016 [100] who evidenced the capacity of the *P. copri* colonization in mice to increase the intraluminal content of succinate, which could be used as substrate for the intestinal gluconeogenesis to then reduce hepatic glucose production via portal glucose sensing.

### 3.3. Mediterranean Diet

The Mediterranean diet is characterized by a high-level consumption of fruits, vegetables, grains, and fish and seafood as the main animal protein [101,102]. Scientific evidence supports that most of the benefits, mainly cardiovascular, of the Mediterranean diet are attributed to the high content of monounsaturated fatty acids (MUFAs), mainly oleic acid; ω-3 polyunsaturated fatty acids (PUFAs), mainly α-linolenic acid, eicosapentaenoic acid and docosahexaenoic acid; polyphenols, flavonoids and non-flavonoids, and fibre [103,104].

Contrary to SFAs, unsaturated fats are associated with metabolic benefits [105,106] and, accordingly, the Mediterranean diet is considered adequate to promote metabolic health without restricting total fat intake. Oleic and α-linolenic acid are GLP-1 secretagogues [107,108,109] and initiate lipid sensing routes to centrally control the endogenous production of glucose [110].

Dietary polyphenols present in vegetables and fruits might also display protective effects against obesity by the modulation of the hypothalamic function. Polyphenols can reach the brain and also initiate gut nutrient sensing routes. In the gut, polyphenols are able to induce the secretion of GLP-1, and PYY [111] in postprandial periods favouring the insulin-mediated glucose-lowering effects [112,113]. Whether or not these effects are mediated by the central nervous system should be further ascertain.

The Mediterranean diet also shapes the gut microbiota and the associated metabolome. Obese individuals adhere to a Mediterranean diet for 2 years show increases in the genera *Bacteroides*, *Prevotella*, and *Faecalibacterium*, and most importantly of *Roseburia* and *Ruminococcus* and the species *Parabacteroides distasonis* and *Faecalibacterium prausnitzii* [114]. Compared with Western diet non-human primate’s consumers, Mediterranean diet consumers also show higher levels of genera *Lactobacillus*, *Clostridium* and *Oscillospira* [115]. The overrepresentation of these gut bacteria species could represent an adaptation of the microbial ecosystem to the higher preabsorptive abundance of PUFAs, polyphenols and complex carbohydrates, not absorbed in the upper gut, and thus able to interact with intestinal bacteria to modulate the pool of nutrient and microbe-derived metabolites activating the gut–brain axis.

Studies in mice reveal that, compared with saturated fats, intake of polyunsaturated fats limit the progression of obesity and induce different changes to the gut microbiota composition [116,117]. Overall lipids are not primarily digested by intestinal microbes but lipid-induced gut microbiota changes can equally influence how the gut senses nutrients. *Lactobacillus* spp. might be overrepresented under a diet rich in linoleic acid since they specifically develop resistance to its toxicity [118] and also use this ω6 fatty acid to produce PUFAs-derived metabolites such as 10-hydroxy-cis-12-octadecenoic acid (HYA) [119]. HYA improves metabolic health, suppressing food intake through lipid sensing-mediated signalling in L cells, in which GPR40 and/or GPR120 activation induces GLP-1 secretion in mice [119]. In addition, gut bacteria might influence lipid sensing routes and the exportation of fatty acids to extra-intestinal tissues by influencing the fatty acid storage in the enterocyte. For instance, *Lactobacillus paracasei* and *Escherichia coli* decrease the intestinal secretion of the absorbed oleic acid but through different metabolites derived from the fermentation of complex carbohydrates [120]. Specifically, intestinal storage of oleic acid is promoted by *L. paracasei*-produced L-lactate by inhibiting malonyl-CoA-induced fatty-acid beta oxidation, a route activated by *E.coli*-produced acetate, which increases oleic acid degradation [120].

Additionally, gut microbiota favours the bioavailability of polyphenols from food in the intestinal lumen through complex multienzymatic reactions [121] modulating the polyphenol-associated gut nutrient sensing. To date, the identification of bacteria species specifically modulating polyphenol metabolic routes is under an early stage of investigation. Nevertheless, some evidence is available indicating, for instance, that *Bacillus subtilis*, which protects against obesity in mice [122], may produce protocatechuic acid from dietary quercetin in the human gut, which can virtually bind to the GLP-1 receptor as predicted by molecular docking simulations [123].

### 3.4. Diets Based on Macronutrients Ratio Variations

Herein we review the effects of diets used for weight loss, which are based on variations of the macronutrients proportions, such as high protein diets and low carbohydrate-high fat diets. We also focus on the potential role of these diets in the postprandial modulation of the gut–brain axis by changes in the pool of microbial and nutritional ligands in the intestinal lumen derived from the dietary macronutrients.

High protein diets are characterized by an increased intake of food rich in proteins (25–35% of energy compared with 12–18% of the standard protein diets) [124] and frequently associated with reduced carbohydrate consumption. These type of diets seem to be appropriate to rapidly induce weight loss [125]. Indeed, high protein diets positively regulate energy metabolism since, compared with other macronutrients, proteins strongly induce satiety and stimulate intestinal gluconeogenesis and thermogenesis [99,126]. Nevertheless, in the long-term, diets with different macronutrients ratios but the same energy-restricted content have similar effects on body weight maintenance, which could also be due to a poor long-term adherence to all diets [127,128,129].

In normal weight or obese human subjects, high protein meals induce the greatest satiety compared with isocaloric diets with high content of carbohydrates or fats, an effect that is dependent on PYY, the secretion of which is preferentially enhanced by proteins [130]. In addition to gut hormones, high protein diets modulate the gut–brain axis to control food intake and energy metabolism by stimulating the intestinal gluconeogenesis from postprandial to postabsorptive periods [99]. Digested peptides in the upper gut antagonize the μ-opioid receptors in the spinal and vagal afferents of the portal vein, a signalling that centrally activates the intestinal gluconeogenesis involved in the glucose sensing-induced food intake suppression [131,132]. Additionally, enriched-protein meals are a source of gluconeogenesis substrates for the gut such as glutamine and glutamate [99]. Compared with carbohydrates and fats, proteins also have the highest effect inducing thermogenesis [126]. Although the underlying mechanisms needs to be clarified specifically for proteins, some studies have identified thermogenesis-dependent gut–brain axis mechanisms mediated by gut hormones. For instance, GLP-1 centrally enhances thermogenesis through sympathetic efferents [133] and the duodenal hormone secretin postprandially activates the thermogenesis to induce satiety [134].

High protein diets also increase the amount of amino acids that can be fermented by gut microbiota in the colon to obtain energy and to produce nutrient sensing ligands. These include amino acid-derived SCFAs, branched chain fatty acids (BCFAs: isobutyrate, 2-methylbutyrate and isovalerate) and other molecules derived from tryptophan or glutamate, among others.

Compared with carbohydrates, the fermentation of proteins produces fewer SCFAs, although it still contributes substantially to microbial organic acid production. Amino acid-derived acetate is produced from glycine, alanine, threonine, glutamate, lysine and aspartate; butyrate is produced from glutamate and lysine and propionate from threonine [135].

Tryptophan is also biochemically transformed by gut microbiota, leading to the production of either serotonin (5-HT), indole, kynurenine or other derivative compounds [136]. Intestinal bacteria modulate the production of 5-HT in the gut directly or indirectly through microbe–host interactions. Members of the gut microbiota also possess tryptophanase activity, mediating the conversion of tryptophan into indole, which serve as interspecies signalling molecule that control bacterial physiology [137]. Indole-producing bacteria include, among others, species belonging to *Bacillus*, *Clostridium*, *Enterococcus*, *Bacteroides*, *Enterobacter*, *E. coli*, *Prevotella*, *Shigella* and *Vibrio* [138]. In addition, the gut microbiota modulates the expression of the rate-limiting host enzyme involved in the conversion of tryptophan to kynurenine, indoleamine 2,3-dioxygenase (IDO1), thus influencing the levels of kynurenine and its derivatives such as kynurenic acid [139]. Although some of these compounds might be toxic, such as indoxyl sulphate and quinolinic acid [140,141], others can potentially activate 5-HT receptors, aryl hydrocarbon receptor (AhR), and GPR35, which affect energy homeostasis (see Section 4 for details).

Intestinal bacteria can also decarboxylase glutamate, producing γ-aminobutyric acid (GABA) via the enzymatic activity of the glutamate decarboxylase, which helps to maintain the intracellular pH of the bacteria [142]. Strains belonging to the genera *Lactobacillus*, *Bifidobacterium*, *Lactococcus*, *Streptococcus*, *Escherichia*, *Listeria*, and *Aspergillus* have been reported to produce GABA [143,144,145], which also modulate nutrient sensing in the gut (see Section 4 for details).

Nevertheless, few studies address associative or causative links between the gut microbiota and high-protein diets on the control of food intake and energy homeostasis via the gut–brain axis.

Overall, diets high in proteins and low in carbohydrates reduce faecal abundance of SCFAs; i.e. butyrate [146], acetate and propionate [147,148], while increasing branch chain fatty acids; i.e., 2-methylbutyrate [146], isobutyrate and isovalerate [53,148,149]. Additionally, high intake of dietary proteins often increases the levels of kynurenic acid and indoxyl sulphate in plasma [150].

Concerning the effects on gut microbiota composition, interventional studies in humans with high protein diets initially suggest that variations (such as reductions of *Bifidobacterium*, *Roseburia* and *Eubacterium rectale*), were a consequence of the reduced intake of dietary fibres or the caloric restriction associated with these interventions [151]. More recent studies comparing the effects of different protein sources suggest that proteins also play a direct role in driving at least microbial-mediated metabolite changes with potential health impacts [146]. This study shows associations between bacterial abundance and faecal metabolites in overweight subjects conducting a 3-week high-protein diet intervention (either soy- or casein-based diet). For example, the study identified a positive correlation between *Oscillospira* and *Odoribacter* and amino acid-derived bacterial metabolites measured by targeted metabolomics and 1H nuclear magnetic resonance [146].

In humans, protein intake is positively associated with ClpB-like gene function [152] and in vitro studies demonstrate that, compared with other macronutrients (D-fructose and oleic acid), *E.coli* requires protein supplementation (bovine serum albumin) to increase the mRNA and protein levels of ClpB, which induces a dose-dependent stimulating effect on PYY secretion [153]. However, future investigations are needed to assess whether postprandial increases in ClpB coupled with bacterial growth account for the PYY-mediated food intake suppression in high protein diets in humans [130].

Larger-scale human interventions are needed to elucidate to what extent changes in microbiota-derived metabolites from dietary proteins affect nutrient sensing and, ultimately, control energy homeostasis, with an especial emphasis on intestinal gluconeogenesis and gut-mediated enhancement of thermogenesis. This will shed light on the link between protein-derived microbial metabolites and high protein-associated weight loss. Furthermore, additional studies are needed to assess the risk and benefits of high-protein dietary interventions in improving metabolic health, since these reduce butyrate production (the main energy source for colonic enterocytes) and increase levels of mucosal and renal toxic compounds (e.g., tyrosine-derivate p-cresol and indoxyl sulphate) [151,154].

Low carbohydrate diets are frequently defined as diets with less than 20% of calories from carbohydrates with high content of fats (55–65%) and occasionally high in proteins (25–30%) [155]. These types of diets include ketogenic diets, frequently characterized by high content of fats, which were originally used to treat epilepsy due to their associated anticonvulsant effect driven by the increased production of ketone bodies and the modulation of GABA neurotransmission and mitochondrial metabolism [156]. Currently, these diets are also conceived for weight loss purposes. The metabolic benefits of ketogenic diets are based on their reduced capacity to postprandially increase glycemia and insulinemia and the concomitant enhancement of cellular catabolic routes using fats as the main source of energy. Accordingly, fat depots are reduced and gluconeogenesis, followed by ketogenesis, are also enhanced to supply glucose to cells [157]. These diets are effective to rapidly lose weight although long-term adherence poses challenges due to their associated contraindications [158]. Some studies reveal that ketogenic diets also induce less hunger and reduce the desire to eat in humans when comparing the appetite assessments before and during adherence to the diet [159]. Low carbohydrate diets also induce satiety in T2D patients [160] and, compared with diets with high content in carbohydrates, have lower capacity to stimulate food intake-related brain areas [161]. Nevertheless, the exact underlying mechanisms of the satiety effects caused by these diets remained elusive, especially those possibly dependent on gut microbiota.

Some studies indicate that diet-induced ketogenesis seems to mediate the reduced circulating levels of ghrelin associated with lower appetite in overweigh/obese individuals following a low energy diet [162] although ketone bodies inhibit the GLP-1 release by the EECs [163] and directly activate orexigenic hypothalamic routes in the brain [164]. Similar to SCFAs, ketone bodies also initiate GPR41 and GPR43 signalling to control energy metabolism. In particular, the acetate binds to GPR43 to induce lipid utilization in plasma [119] and β-hydroxybutyrate antagonizes GPR41 in sympathetic neurons to attenuate the sympathetic mediated metabolism [165]. To date, how these ketone bodies impact on the hypothalamus via the GPR41/43 has not been explored.

Ketogenic diets also change the gut microbiota structure and function in obese individuals with an overall decrease in butyrate and butyrate-producing bacteria, such as *Roseburia* spp. and *Eubacterium rectale* and *Bifidobacterium* spp. These changes are mainly attributed to the reduced content of the diet in complex carbohydrates and the concomitant reduction of SCFAs production [148].

Since ketogenic diets are also diets rich in fats, the identification of the main mechanisms through which these diets impact on the gut–brain axis through gut microbiota-dependent mechanisms is complex. These mechanisms could be related to the reduction of SCFAs generated in the gut by the intestinal microbiota. Nevertheless, the role of other specific gut microbiota adaptations to these diets in shaping the specific pool of bioactive ligands and the existence of potential interactions with the levels of ketone bodies and their effects remain to be investigated.

In this regard, it is unknown whether the ketogenic diet-associated gut microbiota play a causative role in the postprandial control of the gut–brain axis. A recent study conducted in humans and mice identified that, compared with a conventional HFD, a ketogenic diet, high in fats and low in proteins, specifically reduces species from the genus *Bifidobacterium* by a direct effect of intestinal ketone bodies inhibiting their growth [166]. Additionally, the ketogenic diet displays metabolic improvements in mice, although these were attributed to a protective effect on the intestinal immunity rather than to the modulation of the gut–brain axis [166]. A direct role of ketogenic diets modulating the gut–brain axis was evidenced in another study in mice demonstrating that a carbohydrate-restricted diet with high content of fats favours cross-feeding between *Akkermansia muciniphila* and *Parabacteroides*, resulting in a reduced gamma-glutamyltranspeptidase activity in faeces driving an increased GABA/glutamate ratio in the hippocampus that in turn confers protection against refractory epilepsy [167]. Although how ketogenic diets centrally control energy homeostasis through gut microbiota-mediated mechanisms remains uncertain, given the role of GABAergic neurotransmission on the central control of energy homeostasis and food intake (see Section 4 for details) it seems plausible that ketogenic diet modulates the gut–brain axis in postprandial periods by a similar gut microbiota-dependent mechanism operating in the hypothalamus. On the other hand, the gut microbiota, by modulating the lipid metabolism, seems to influence the circulating levels of β-hydroxybutyrate, which exerts neuroprotective effects on the brain, although this effect has not been explored for its relationship with metabolic health [168].

## 4. Microbial Ligands Mediating Gut–Brain Communication and Energy Homeostasis

**Figure 1:**
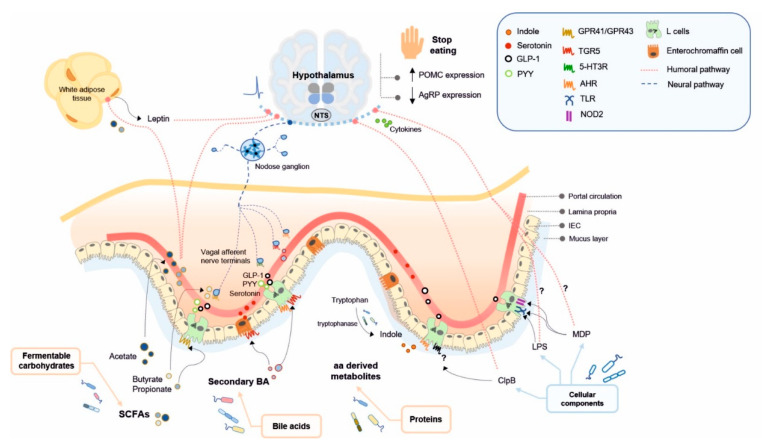
Bacterially produced metabolites from dietary nutrients and structural components of non-dietary origin modulate food intake in the brain (hypothalamus) through humoral and/or enterodocrine and neural signalling pathways. Here, we represent the pathways by which bacterial metabolites and non-dietary bacterial components (LPS, MDP and ClpB) induce an anorexigenic response in postprandial periods and a long-term food intake control. 5-HT3R, 5-hydroxytryptamine type 3 receptor; aa, amino acid; AgRP, agouti gene-related peptide; AHR, aryl hydrocarbon receptor; BA, bile acids; ClpB, caseinolytic peptidase B; GLP-1, glucagonlike peptide-1; GPR41/FFAR3, free fatty acid receptor 3; GPR43/FFAR2, free fatty acid receptor 2; IEC, intestinal epithelial cells; LPS, lipopolysaccharide; MDP, muramyldipeptide; NOD2, Nucleotide-binding oligomerization domain 2; NTS, nucleus tractus solitarius; PYY, peptide YY; POMC, proopiomelanocortin; SCFA, short-chain fatty acids; TGR5, takeda G protein-coupled receptor 5; TLR, Toll-like receptor.

Here, we review the microbial products, including bacterial metabolites and bacterial cell components, that might impact on brain functions by modulating nutrient sensing signalling through enteroendocrine humoral and neural pathways, and could contribute to controlling energy homeostasis (Figure 1, Table 1). Beyond the control of food intake, gut microbiota might influence the whole-body energy metabolism by modulating the parasympathetic and sympathetic efferent tone [47,169], although this mechanism has been the subject of far fewer studies. Thus, this section focuses on the role of gut microbiota in controlling food intake and energy homeostasis, mainly through effects on the hypothalamus-mediated food-intake suppression and, specially, in the postprandial periods.

**Table 1:** Main microbially derived ligands of dietary and non-dietary nature involved in gut-to brain nutrient sensing and control energy homeostasis.

Dietary Nutrients	Gut Bacterial-Derived Ligand	Bacterial Producers	Bacterial-Producing Enzyme	Receptor	Pathway	Function	References
**Fermentable carbohydrates**	SCFAs (acetate, propionate, butyrate)	*Prevotella* [90], *Ruminococcus* [90], *Bifidobacterium* sp. [91], *Prevotella* [95,96]	Phosphate acetyltransferase and acetate kinase for acetate	FFAR2/GPR43 (L cells)	Humoral pathway	Food intake suppression, ARC neuronal activation, increase in acetyl-CoA carboxylase activity and AMPK inducing an increase in POMC and reduction in AgRP expression, leptin release from adipocytes	[89,170,171,172,173]
			Enzymes involved in succinate, acrylate and propanediol pathways for propionate	FFAR3/GPR41 (L cells, enteric neurons, nodose ganglion neurons)	Humoral pathway, gut nutrient sensing pathways (GLP-1, PYY)	Food intake suppression, leptin release from adipocytes, control of postprandial glucose, control of intestinal gluconeogenesis	[89,132,173,174,175,176,177]
			Phosphate butyryltransferase and butyrate kinase for butyrate	FFAR3/GPR41 (L cells, enteric neurons, nodose ganglion neurons)	Gut nutrient sensing pathways (GLP-1, GIP, vagal afferents)	Food intake suppression, stimulation of POMC expression, suppression of AgRP expression, suppression of orexigenic neurons activity	[89,178,179,180,181]
**Bile acids (BAs)** **(involved in lipid digestion)**	Secondary BAs	Members of the genera: *Lactobacillus* [182,183,184], *Bifidobacterium* [182,185], *Enterococcus* [186,187], *Clostridium* [182,188], *Listeria* [182,189], *Bacteroides* [182]	Bacterial bile salt hydrolases (BSH) (deconjugation of primary BA to secondary BA)	TGR5 (L cells, vagal afferents, nodose ganglion neurons, hypothalamic neurons)	Humoral pathway, gut nutrient sensing pathways (GLP-1, PYY, 5-HT, vagal afferents)	Food intake suppression in synergy with CCK1R activation, activation of POMC/CART-expressing hypothalamic neurons, glucose homeostasis, 5-HT3R activation in intestinal vagal afferent terminals (probably modulating food intake)	[190,191,192,193,194,195,196,197,198,199,200]
**Proteins**	Indole	Members of the genera: *Bacillus*, *Clostridium*, *Enterococcus, Bacteroides*, *Enterobacter*, *Escherichia*, *Prevotella*, *Shigella* and *Vibrio* [138]	Tryptophanase (tryptophan to indole)	AHR (L cells)	Gut nutrient sensing pathways (GLP-1)	Contribution to eating patterns unknown	[136,201,202,203]
	GABA	Members of the genera: *Lactobacillus*, *Bifidobacterium*, *Lactococcus*, *Streptococcus*, *Escherichia*, *Listeria*, and *Aspergillus* [143,144,145]	Glutamate decarboxylase (glutamate to GABA)	GABA_A_, GABA_B_ (L cells, vagal afferents)	Gut nutrient sensing pathways (potentially through vagal afferents)	Contribution to nutrient sensing in the brain unknown	[142,204,205,206,207]
**Bacterial cellular components**	ClpB (mimetic of α-MSH)	Order *Enterobacteriales*, including *E. coli* strains and *Hafnia* genus [208]	-	Unidentified	Humoral pathway, gut nutrient sensing pathways (PYY)	Food intake suppression by increasing POMC and decreasing AgRP expression, enhancement of POMC neuronal activity	[41,42,154,209,210,211]
	LPS	Gram-negative bacteria [212]	-	CD14/TLR4 (enteric neurons, nodose ganglion neurons)	Humoral pathway, gut nutrient sensing pathways (GLP-1 and potentially through vagal afferents)	Reduction of food intake, enhancement of GLP-1-induced NO production in enteric neurons (possibly contributing to an anorexigenic shift in neuropeptides expression), satiogenic effect probably by changes in hypothalamic cytokine expression, increase nodose ganglion neurons excitability	[43,45,47,213,214,215,216,217,218]
	MDP	Gram-positive bacteria (minor component in Gram-negative bacteria) [219]	-	CD14/NOD2/TLR2 (L-cells, enteric neurons)	Humoral pathway, gut nutrient sensing pathways (GLP-1)	Reduction in food intake, enhancement of GLP-1-induced NO production in enteric neurons, glucose tolerance	[43,45,47,213,214,215,216,217,220]

### 4.1. Short Chain Fatty Acids

The function of SCFAs in protecting against metabolic alterations of diet-induced obesity is relatively well-established. Supplementing diet with fermentable carbohydrates, such as inulin, oligofructose or pectin, reduces food intake and improves HFD-induced glucose intolerance and weight gain in rodents and humans [170,221,222,223,224,225]. These effects are associated with SCFA production.

Although the three main SCFAs activate both GPR43/FFAR2 and GPR41/FFAR3, acetate is a potent agonist of FFAR2 [226], whereas propionate and butyrate have higher affinity for FFAR3 [226]. Both receptors are expressed in L-cells [227,228], and FFAR3 has been detected in enteric neurons [229] and in nodose ganglia cells [230]. Besides being agonists of FFAR2 and FFAR3, all SCFAs and lactate can potentially affect food intake by inhibiting the signalling of the orexigenic hormone ghrelin. In Hek293a cells stably expressing human growth hormone secretagogue receptor type 1a (GHSR1a), the three SCFAS (acetate, propionate, butyrate) and lactate reduced the Ca^2+^ influx in presence of ghrelin and showed antagonistic GHSR-1a properties as they attenuated the ghrelin-mediated receptor internalization [231]. As this SCFAs-mediated signalling is still under an early stage of investigation (revised by [232]), here we focus on the role of SCFAs in food intake suppression, acting as FFAR2/FFAR3 agonists.

SCFAs are known to regulate food intake by modulating hypothalamic function, either reaching systemic circulation to the brain or via nutrient signalling mediated directly by GLP-1 and PYY generated in EECs or via vagal afferents. Among SCFAs, acetate seems to reach the brain through systemic routes, while propionate and butyrate mainly activate gut nutrient sensing pathways.

The analysis of biodistribution of 11C-acetate infused in the mouse colon indicates that acetate crosses the blood–brain barrier and reaches the hypothalamus, suppressing food intake short-term [171]. Moreover, acetate from fermentable fibre increases the hypothalamic neuronal activation in the ARC, but not in the ventral medial hypothalamus (VMH) or the PVN [170,171]. These changes are accompanied by an increase in hypothalamic activity of acetyl-CoA carboxylase and AMP-activated protein kinase (AMPK), subsequently inducing a downstream acute rise in POMC expression and reduction in AgRP expression [171,172]. In addition, systemic acetate enhances leptin release from adipocytes, possibly via an as yet undetermined FFAR2-dependent mechanism [173], thus contributing to a shift towards an anorexigenic neuronal activation pattern in the ARC [233]. Contrary to the abovementioned control diet-based research, a study in rats indicates that, under an energy-dense diet, gut microbiota enhance the production of acetate from glucose and fatty acids, contributing to diet-induced obesity [234]. Indeed, HFD increases acetate levels in the intestinal lumen and also systemically results in hyperphagia and an enhanced parasympathetic tone in β-cells, which impairs glucose stimulating insulin secretion [234]. The role of acetate in signalling through gut nutrient sensing routes is less evident. Some studies reveal that acetate does not stimulate GLP-1 or PYY secretion [171,235] and others report acetate-induced PYY secretion in the distal, but not proximal colon in a small cohort of obese humans without exploring its effects on food intake [236,237].

In overweight humans, the acute administration of inulin-propionate ester, which enables propionate delivery in the colon specifically, increases the postprandial secretion of GLP-1 and PYY along with reduced food intake, together with a non-significant trend to decrease long-term energy intake [225]. In mice, propionate also robustly stimulates both GLP-1 and PYY secretion either in vivo or in primary murine colonic cultures, probably via a FFAR2-dependent mechanism [174]. Additionally, propionate acts as a food intake suppressor through independent mechanisms of the endocrine actions of gut hormones. Indeed, independently of changes of the circulating levels of GLP-1 and PYY, non-obese healthy men receiving inulin-propionate showed reduced *ad libitum* food intake and lower oxygen level-dependent signal in reward-related brain structures [238]. Thus propionate can impact on brain function through vagal afferents innervating the gut and the portal vein. In this regard De Vadder and colleagues demonstrated that luminal propionate controls the postprandial levels of glucose through a gut–brain loop [175]. In particular, this process is initiated by the propionate-induced activation of FFAR3 expressed in the afferents of the portal vein, which leads to the activation of neurons in brain regions receiving vagal and spinal inputs, i.e., DVC, spinal C1 segment and the parabrachial nucleus (PBN), respectively, as well as in the hypothalamic areas receiving inputs from DVC and PBN, i.e., PVN, the lateral, hypothalamus (LH) and the ARC. Apparently, propionate centrally controls the intestinal gluconeogenesis, a process that induces metabolic benefits [132,176], including the reduction of the endogenous production of glucose independently of insulin [175].

Furthermore, like acetate, elevated plasma propionate levels induce leptin release from adipocytes via the somewhat controversial FFAR2- or FFAR3-dependent mechanism [173,177], promoting hypothalamic anorexigenic neuronal activation [233].

Among the three main SCFAs, butyrate may be the strongest stimulator of anorexigenic peptides [178] and the most potent suppressor of food intake [179]. In obese and healthy humans, all three main SCFAs separately administered increase plasma levels of PYY in both fasting and postprandial conditions [236]. In mice, oral, but not intravenous, administration of butyrate reduces food intake, according to decreased neuronal activity in NTS and DVC [180]. Additionally, butyrate influences the hypothalamic circuitry, suppressing the activity of orexigenic neurons [180], decreasing AgRP expression [181] and enhancing POMC expression [181]. In addition, subdiaphragmatic vagotomy eradicates the anorectic effects of butyrate, suggesting that the vagal nerve is necessary to convey the short- and long-term satiety signalling of acute or chronic administration of butyrate in the context of obesity [180]. Moreover, intraperitoneal butyrate induces activation of nodose ganglia neurons [179]. Intestinal butyrate may transmit satietogenic signals by stimulating GLP-1 and GIP secretion in L-cells and K-cells, respectively [178], and these effects are probably reinforced by ghrelin inhibition [181]. It is not clear whether butyrate induces PYY secretion, since studies report no changes [181], decrease [235] or slight increase [178] in PYY release as a result of butyrate increase in the caecal content of animals orally receiving a probiotic, a pea protein supplementation or oral butyrate administration. Furthermore, it is not yet clear whether butyrate-induced gut hormone secretion is mediated by a FFAR3-dependent mechanism [178,181]. Therefore, the evidence about the mechanism through which SCFA are involved in satiety signalling is not fully consistent, probably due to experimental differences, for instance, in hormone measurement timings, types of dietary fibre supplementation, administration site and the use of different animal models.

### 4.2. Microbial Metabolites of Bile Acids

Bile acids (BA) are steroid acids synthesized in the liver from cholesterol, conjugated to either taurine or glycine and postprandially released in the duodenum to facilitate the absorption of dietary lipids and fat-soluble vitamins [239,240,241]. The majority of primary BA secreted in the intestine are actively reabsorbed in the ileum and transported back through the portal circulation to the liver (enterohepatic circulation) [242]. The remaining small portion of primary BA are deconjugated and dehydroxylated in the ileum and colon by intestinal bacteria into secondary BAs [243,244], mainly DCA and lithocholic acid (LCA) in humans and rodents, and murideoxycholic acid in rodents [239,245]. Bacterial bile salt hydrolases (BSH) are essential enzymes for deconjugation of primary BA to secondary BAs [190]. In humans, BSH genes and enzymes have been characterized in Gram-positive bacterial species of the genera *Lactobacillus* [182,183,190], *Bifidobacterium* [184,190], *Enterococcus* [185,186], *Clostridium* [187,190] and *Listeria* [188,190] and in Gram-negative bacteria of the genus *Bacteroides* [189,190].

Besides their role in lipid digestion, BAs have recently been characterized as ligands of takeda G protein-coupled receptor 5 (TGR5), regulating lipid and glucose metabolism once activated [246,247,248]. Dietary macronutrients and feeding patterns influence the composition and secretion of bile acids, respectively, and thus the microbiota-mediated bile acids’ effects on the host. Interventional studies in humans reveal that a fibre-rich low-fat diet is associated with low levels of all secondary BAs, and both glycine- and taurine-conjugated bile acids in faeces [249]. By contrast, a high fat diet increases the abundance of unconjugated and secondary BAs (DCA, TDCA, 12keto-LCA, 3b-DCA and TLCA) coupled with changes in species belonging to the genera *Bacteroides*, *Clostridium*, *Bifidobacterium* and *Lactobacillus* and a positive correlation between *Bacteroides* and DCA [250].

Studies in humans and rodents report that microbial BAs modulate energy homeostasis directly by reaching the brain through systemic circulation and/or activating gut nutrient sensing routes. The main functional effects of BAs reported are on feeding behaviour or glucose homeostasis. Plasma BAs levels notably rise postprandially in humans [251,252] and rodents [253], thus fluctuating in the systemic circulation along with the circadian rhythm upon food intake, suggesting a role of BA as lipid-sensing molecules and short-term satiety signals that reach the brain through a humoral route. Diet fat content is positively associated with plasma BAs concentrations in humans [252] and mice [191], and with hypothalamic BAs concentrations in mice [191]. Interestingly, DCA and other BAs have been detected in the rat brain [254,255], and their levels are positively correlated with plasmatic levels [254], indicating that secondary BAs may reach the brain from the intestine by diffusion across the blood–brain barrier [254]. Accordingly, TGR5 expression has been detected in neurons [192] and more recently in the hypothalamus related to glucose metabolism [191]. Altogether, this evidence suggests secondary BAs generated by gut microbiota activity potentially play a role in the direct control of hypothalamic function.

In addition, secondary BAs activate enteroendocrine and/or neural gut nutrient sensing routes. Studies in mice demonstrate that downregulation of TGR5 in the context of diet-induced obesity impairs glucose tolerance [193] and increases food intake coupled with a substantial decrease in the plasma levels of GLP-1 and PYY [194,195]. By contrast, TGR5 overexpression in mice improves oral glucose tolerance by inducing a marked enhancement of the postprandial secretion of GLP-1, probably due to increased BAs flow after a highly lipidic meal, [193]. Gut bile acid sensing is also supported by other in vitro [196] and in vivo [195,197] studies in mice showing that secondary BAs trigger GLP-1 and PYY secretion from EECs via TGR5 activation. Human colonic GLP-1-producing EECs also express TGR5, the activation of which by TDCA increases the secretion of GLP-1 [256]. Importantly, obese subjects with or without diabetes show an impaired BA metabolic pathway [256] which might contribute to aberrant GLP-1 secretion [257]. Similarly, experiments in mice demonstrate that secondary BAs induce the secretion of 5-HT [198] subsequently activating 5-HT3 receptors on vagal afferent terminals [199]. The impact of secondary BAs resulting from gut microbiota activity on the brain is still understudied [247] but secondary BAs, through TGR5-GLP-1 and/or 5-HT gut sensing pathways, can potentially modulate food intake and energy homeostasis via efferent routes. Specifically, the role of secondary BAs in inducing satiety through TGR5-activation in vagal afferent neurons has been demonstrated. Indeed, TGR5 expression has been detected in the nodose ganglia cells colocalizing with CCK-1R in rats [200]. Moreover, vagal TGR5 mediates DCA-specific activation of POMC and CART-expressing neurons, but not orexigenic neurons, in the hypothalamus resulting in reduced spontaneous postprandial food intake [200]. Additionally, silencing TGR5 and CCK-1R in the nodose ganglia has an additive effect increasing spontaneous food intake and suggesting a synergistic effect of BA and CCK in hypothalamic short-term satiety signalling via the vagal pathway [200].

Therefore, the bacterial BAs bioconversion capacity may have a strong impact on the intestinal availability of secondary BAs [258,259]. Subsequently, the postprandial enhancement of the TGR5-mediated signalling may be affected by these secondary BAs generated in the intestine as well as by the BAs pool that reaches the brain [247], contributing to the modulation of hypothalamic function. However, to our knowledge, there are currently no studies reporting the effects of specific bacterial species or strains on BA sensing to centrally control energy homeostasis.

### 4.3. Amino Acid Derived Metabolites

In the gut, as well as in the brain, 5-HT is synthesized from tryptophan [260], an essential amino acid obtained from dietary proteins [261]. Studies in rats demonstrate that 5-HT is released by enterochromaffin cells (ECs) in response to nutrient stimuli such as carbohydrates, glucose or lipids [262,263]. 5-HT binds to 5-HT3 receptors in the vagal afferents, acting thus as sensory transducers [262]. Functionality, the activation of 5-HT signalling in vagal afferents mediates glucose induced inhibition of gastric emptying and lipid-induced food intake suppression, which occurs simultaneously with CCK1 signalling [263,264]. Similarly, in humans, inhibition of 5-HT3 receptor increases the liquid meal ingested [265].

*Hafnia alvei*, *E. coli* K-12 and species and strains of the genera *Lactococcus*, *Lactobacillus*, *Streptococcus* and *Klebsiella* have been shown in vitro to synthesize 5-HT from tryptophan [266]. The mechanisms by which gut bacteria directly synthesize 5-HT are not clearly established as bacteria lack tryptophan hydroxylase 1 (TPH1), an essential enzyme for 5-HT biosynthesis. Therefore, an alternative mechanism has been proposed, in which tryptophan is decarboxylated to tryptamine [267]. The role of gut microbiota as an enhancer of 5-HT biosynthesis and release from ECs may partly occur through host–microbe interactions mediated by bacterial structural components or metabolites according to certain reports. For example, *Escherichia coli* modulates the host tryptophan hydroxylase-1 activity, enhancing the synthesis of the 5-HT precursor 5-hydroxytryptophan (5-HTP) and the extra-cellular levels of 5-HT through interaction with secreted host derived factors [268]. This strain also contributes to 5-HT clearance by its uptake into enterocytes through the serotonin transporter SERT, which is required for completion of the serotonin circuit synthesis. Other studies show that spore-forming microbes from a healthy human increase 5-HT in colon and blood, through a process that seems to involve host interactions with bacterially produced metabolites such as deoxycholate and SCFAs [269,270]. Probably acting through SCFAs, gut microbiota upregulates the expression of THP1 in colonocytes as demonstrated by the colonization of germ-free mice with mouse or human gut microbiota [270].

A recent study reveals that ECs differentially express chemical sensors according to their spatial location in the gut. Surprisingly, in the small intestine, ECs do not express nutrient sensors but might indirectly release 5-HT in response to nutrient stimuli through the paracrine action of GLP-1, from the neighbour EECs [271]. By contrast, compared with small intestine ECs, colonic ECs present higher expression of microbial sensors such as TGR5, which binds to secondary BAs; GPR132, to acyl amides and lactate; GPR35, to aromatic acids, OLFR558, to isovalerate and FFAR2, to SCFAs, all able to trigger 5-HT release [271]. Accordingly, gut microbiota, through their metabolites, would induce the release of 5-HT in colon and indirectly in the small intestine by stimulating GLP-1 secretion. In fact, although 5-HT cannot cross the blood–brain barrier [139], peripheral 5-HT has been demonstrated to reduce food intake [272,273,274], suggesting an alternative signalling pathway for the effects of gut-derived 5-HT on the regulation of appetite [275,276].

Studies indicate that, in contrast to 5-HT, its precursor 5-HTP can cross the blood–brain barrier, probably leading to 5-HT production in the brain [139,277,278] and accounting for the role of gut-derived 5-HTP in eliciting satiety. In fact, 5-HTP biosynthesis is enhanced in ECs by gut microbiota-derived SCFAs by upregulating expression of TPH1 as indicated above [278]. Thus, these findings support a role of the gut microbiota-induced 5-HT and 5-HTP in food intake, although further investigations are needed to specifically identify the postprandial microbially produced metabolites and structural products potentially influencing feeding behaviour via this route.

Tryptophan can be fermented into indole [201] which is a ligand of aryl hydrocarbon receptor (AHR), a transcription factor that regulates gene expression. In vitro experiments with GLUTag cell lines show that activation of AHR with an agonist increases the expression of proglucagon as well as GLP-1 secretion [202]. In line with this, experiments with primary mouse colonic L cells demonstrate that indole elicits rapid GLP-1 release during short exposure but that release is inhibited over longer periods and at lower doses [203]. How these actions are coordinated with eating patterns remains unknown; however, the authors proposed that indole at low concentration suppress GLP-1 from L colonic cells but induces an opposite effect in response to high-protein diet. Although indole-mediated nutrient sensing signalling is in an early research stage, the evidence above suggests that AHR might act as a sensor of microbially produced metabolites capable of triggering GLP-1-mediated nutrient signalling from the L cells of the gut to the brain.

In mice, obesity has been associated with increased intestinal IDO1, an enzyme catalysing tryptophan degradation via the kynurenine pathway, thus limiting the bacterial production of indole from tryptophan and increasing kynurenine and its derivatives such as kynurenic acid [279]. In mice, intraperitoneal administration of kynurenic acid induces energy expenditure without affecting locomotion or food intake [280]. This effect seems to be dependent on the activation of GPR35 in the adipose tissue [280], a receptor that has also been detected in sensory neurons of the extrinsic intestinal innervations [281], suggesting that kynurenic acid could play a role in gut-to-brain sensory transmission, which is as yet unexplored.

To our knowledge, so far, studies are lacking on the impact of GABA produced by gut microbes from the dietary amino acid glutamate on the gut–brain communication and, thus, on the control of energy homeostasis. Therefore, the pathways by which microbially produced GABA may influence the hypothalamus function can only be hypothesized from unconnected observations. GABA is the main inhibitory neurotransmitter in the central nervous system. Peripheral GABA cannot cross the blood–brain barrier [282], but activates gut nutrient sensing signalling pathways. First, GABA has been demonstrated to stimulate GLP-1 release from the EECs line GLUTag [204]. Moreover, GABAB receptors are expressed along the gastrointestinal tract [283,284], and co-localized with 5-HT-producing cells identified as morphologically similar to EECs [284], probably inhibiting 5-HT-release by ECs [285]. Second, GABA transmits sensory information by activating GABAA [205] and GABAB receptors [206] expressed in vagal afferents or by secreting exosomes from GABA-stimulated intestinal cells that, in turn, activate neurons [207]. Nonetheless, specific studies are needed to understand the contribution of gut microbiota-derived GABA to nutrient sensing from the gut to the brain and, thus, to the control energy homeostasis in both lean and obese subjects.

### 4.4. Cellular Components of Gut Bacteria

Certain cellular components of intestinal bacteria stimulate gut-to-brain routes of communication, which may be involved in the central control of energy homeostasis, especially through food intake modulation. This is the case for protein fragments of gut bacteria displaying molecular mimicry with human appetite-regulatory peptides and neuropeptides; i.e., protein fragments of the human α-MSH sequence being identical to gut bacterial-derived proteins from *E. coli, Bifidobacterium longum*, *Bacillus cereus*, and certain enteropathogenic bacterial strains [286]. In particular, ClpB produced by *E. coli* K12 is a mimetic of the anorexigenic peptide α-MSH [41], which has a discontinuous five-amino acid overlap containing part of the α-MSH sequence [287]. ClpB is a heat-shock protein with ATPase and chaperon activity mediating the resolubilization of heat-denaturated protein aggregates, thus having a protective function for bacterial cell-induced damage. More recently, the α-MSH-like motif identified within ClpB protein has been confirmed to be specific to the order Enterobacteriales and conserved among its taxa, including *E. coli* strains and *Hafnia* genus [209].

Recent preclinical and clinical studies have demonstrated the effectiveness of ClpB protein on modulating energy homeostasis. In humans, shotgun metagenomic analysis of faeces revealed that gut microbiota ClpB gene, which is associated with decreased weight gain [152], was lower in obese compared to lean individuals [152]. In obese mice, oral administration of the native *E.coli* K12 strain, unlike the ClpB-deficient strain, decreased body weight gain [209], while its food-intake suppression activity was lost when the ClpB-deficient *E. coli* was administrated [41]. In addition, intragastric administration of *Hafnia alvei* HA4597, a ClpB-producer, reduces caloric intake and increases lipolytic effects in *ob*/*ob* mice coupled with reduced fat mass and body weight gain in both genetically and diet-induced obese mice [209]. Along with the aforementioned effects, Lucas et al. found a decrease in glycaemia, plasma cholesterol and alanine aminotransferase, a marker of obesity-induced steatohepatitis [208]. Mechanistically, ClpB modulates the hypothalamic circuit that controls food intake. In particular, AgRP expression is decreased in obese mice administered the ClpB-producer *H. alvei* [209], while in an animal model of anorexia increased ClpB plasma levels were associated with increased POMC expression [210]. Moreover, *bdnf* mRNA levels increased in mice receiving *E. coli* proteins collected at stationary phase, which include ClpB [42]. These findings prove the contribution of ClpB in upregulating the expression of hypothalamic anorexigenic neuropeptides.

In addition, it is known that ClpB transmits nutrient information from the gut to the brain, directly reaching the hypothalamus when traveling in the systemic circulation, or indirectly by the activation of gut nutrient sensing signalling. The presence of ClpB has been reported in the plasma and in the hypothalamus of rodents and healthy humans [211,288]. Moreover, ClpB protein plasma levels in obese mice are increased by treatment with the ClpB-producer *H. alvei* HA4597, in accordance with food intake reduction [209] and patients with eating disorders show elevated ClpB plasma concentrations compared to healthy individuals [288]. Ex vivo electrophysiological experiments also reveal that ClpB from the stationary stage of *E.coli* directly stimulated the firing rate of POMC neurons, while systemic administration of *E.coli* in the stationary stage increased cFos immunolabelled POMC neurons in the ARC and VMH [42].

ClpB has also been found in the colon of mice, rats, and healthy humans [211]. ClpB stimulates PYY secretion in cultured rat intestinal mucosa in a dose-dependent manner [211] and the ClpB-induced PYY secretion may be enhanced by nutrient-induced bacterial growth [42]. Accordingly, in response to food intake, ClpB initiates a PYY-mediated endocrine or neural nutrient sensing signalling that ultimately regulate the hypothalamic function in suppressing food intake. However, the receptor linking the ClpB agonist with PYY secretion has yet to be identified [153].

A synthetic fragment of ClpB has partial agonist activity on MC3R and MC1R, but not on MC4R [287], whose activation by α-MSH induces PYY secretion [289]. However, the ability of the ClpB-α-MSH-like motif to activate MCRs [287] supports a spatial complementarity of bacterial-derived ClpB and MCRs and, therefore, further investigations regarding the receptors by which ClpB drives its satiating effects are required.

Overall, ClpB is suggested to have a satietogenic effect by systemic and/or neural/endocrine routes. By a systemic route, plasmatic ClpB depends on the quantity of ClpB-producing bacteria in the intestine and, therefore, plasmatic ClpB might be a long-term satiety signal by modulating hypothalamic neuropeptide expression, ultimately influencing meal patterns. By a neural/endocrine route, ClpB might also be a short-term meal termination signal by stimulating release of satiety hormones in the intestine.

Some studies also indicate that LPS and MDP may play a role as food intake modulators. LPS is the outer membrane’s major component of Gram-negative bacteria [290]. In obesity, LPS plasma levels are increased due to elevated gut permeability [212], leading to the low-grade inflammation characterizing this metabolic disorder [291]. MDP is a minor component of the peptidoglycan of the cell wall of Gram-negative and more abundant in Gram-positive bacteria [219]. Under normal conditions, MDP is released constantly from degraded gut bacteria [45]. LPS and MDP are CD14/TLR4 and NOD2 agonists, respectively, and both enhance GLP-1-induced nitric oxide production in enteric neurons [47], which may contribute to satiety signalling to the hypothalamus by promoting a shift towards anorexigenic neuropeptides expression [213]. Moreover, MDP activates NOD2 expressed in L-cells to induce GLP-1 release in healthy, but not diet-induced obese mice [220]. Although these effects have been related to glucose tolerance [220] MDP-induced GLP-1 release should also be studied for its effects on food intake.

Interestingly, several studies with rodents report clear effects of LPS and MDP on satiety. Acute or chronic intraperitoneal injections of LPS or MDP reduce food intake [43,45,214,215]. Some studies have found LPS to induce stronger anorexigenic effects in the brain compared to MDP [44,216,292]. The satiety-producing potential between both bacterial components is probably explained by their different abilities to enhance cytokine expression in the hypothalamus [216], since no changes have been detected in the expression of the neuropeptides POMC, NPY or leptin after LPS or MDP administration to the brain [216].

The anorexigenic effects of LPS and MDP seem to be mediated by CD14 for both and by TLR4 for LPS and TLR2 for MDP [217]. The LPS from *E. coli* isolated from healthy humans has been demonstrated to increase murine nodose ganglia neurons excitability in vitro via a TLR4-dependent mechanism [218], suggesting that the intestinal-derived LPS effects on hypothalamic regulation of food intake is mediated by vagal afferent neurons. In contrast to these findings, subdiaphragmatic vagal deafferentation in rats did not suppress food intake reduction after intraperitoneal injection of LPS or MDP, suggesting that peripheral administration of both bacterial components enhances appetite reduction via a humoral but not vagal pathway [214]. Moreover, MDP gavage in rats has no effects in MDP circulating levels nor in feeding patterns, while intraperitoneal MDP reduces food intake, indicating the importance of the humoral pathway for MDP satiety signalling [45]. However, intestinal MDP translocation should be specifically investigated to better define the underlying mechanisms of intestinal-derived MDP on appetite regulation.

Lugarini et al. found that LPS and MDP suppress food intake in obese rats to a similar extent as in non-obese rats [215], suggesting that both bacterial components exert anorexigenic effects regardless obesity. However, the doses of injected LPS and MDP in this study may not reflect the amount of LPS and MDP crossing the intestinal epithelial barrier, whose integrity depends on the metabolic state [212]. Further investigation is required to fully unravel the mechanisms and pathways through which intestinal structural bacterial components reach the hypothalamic centre of appetite regulation under healthy and pathological conditions in vivo.

## 5. Tackling Obesity with Gut Microbes Mediating in Gut–Brain Communication

Hyperphagia and obesity are caused by defective sensitivity in the hypothalamus or in food reward brain areas to peripheral signals reflecting the nutritional status of the body. Current antiobesity drugs mainly target the central nervous system to suppress appetite. Monotherapies including the agonist of 5-HT2C receptor, lorcaserin, or the protease-resistant long-acting GLP-1, liraglutide, as well as combinational therapies of stimulators of noradrenaline or serotonine-release combined with sympathomimetic anticonvulsant or opiod receptor antagonist are currently approved for treating obesity and T2D, combined with exercise and a balanced diet [14,15,293]. As emerging pharmacotherapies, gut peptides acting as potential agonists of lipid sensing and bile acids receptors and, thereby, as secretagogues are being preclinically and clinically tested as potential drugs to improve metabolic disturbance, especially T2D [294]. Notably, bariatric surgery is the most effective therapy for treating obesity, producing important and sustained weight-loss. This is conducted only in a limited number of subjects with a body mass index (BMI) > 40 and also implies surgical risks [295,296]. It is hypothesized that, rather than mechanical alterations, bariatric surgery alters gut signals sensed by the brain, including amplification of postprandial secretion of gut hormones, resulting in a beneficial impact on energy homeostasis [13]. Research is underway to advance the clinical management of obesity with less invasive pharmacotherapies, which mimic the molecular adaptations of bariatric surgery, including those based on gut–peptide combinatorial strategies [13]. However, the applicability of new drugs is frequently limited by unwanted side effects and safer alternatives are being considered, such as devices to modulate the vagal nerve [14].

In the light of this review, the gut microbiota represents a potential target for favourably regulating gut–brain communication and thereby controlling food intake, energy homeostasis and obesity. Indeed, the supplementation of key intestinal bacteria could increase the microbiota-derived bioactive molecules acting as enhancers of gut hormone secretion and as vagal afferent stimulators, optimizing the response to nutritional clues via gut–brain signalling in obesity. Notably, promising studies have revealed that the gut microbiota might act in harmony with the postprandial processes in the gut that control food intake, as occurs for bacterial-produced ClpB.

Currently, microbiome-based approaches, including faecal microbiota transplantation (FMT) and the administration of intestinal bacteria (probiotics or bio-therapeutic products), are being explored for their impact on gut microbiota structure and function for therapeutic or preventive purposes.

The clinical applicability of FMT to treat obesity is being explored in experimental clinical trials. To date, a couple of studies have been completed and indicate that the microbial shift towards a lean-like profile induced by the oral administration of capsules containing the faecal microbiota from lean donors was not associated with metabolic benefits in the recipients [297,298]. By contrast, an improvement in insulin resistance was observed in male volunteers with metabolic syndrome after a duodenal infusion of gut microbiota from lean donors obtained from small intestine biopsy species [299]. Nevertheless, investigations in this field provide limited data regarding how the microbiota-based approaches specifically control the gut–brain axis in obesity [300].

New indigenous intestinal bacterial species are also being identified and tested for their potential use as next generation probiotics or live biotherapeutic products [301] to maintain metabolic health and combat obesity. This strategy may also contribute to progressing towards the development of safer and more effective microbiome-based strategies for the clinical management of obesity as compared to FMT [302]. Some examples of these new bacterial species and strains are *Eubacterium hallii* L2–7, *Akkermansia muciniphila* ATCC^®^ BAA-835™ or *Bacteroides uniformis* CECT 7771. These were initially selected based on associations between an increased abundance of the bacterial species and a healthy metabolic phenotype in a substantial number of human studies. To date, the clinical efficacy in overweight volunteers has already been demonstrated for *A. muciniphila* (clinical trial no. NCT02637115). The pasteurized *A. muciniphila* significantly improved metabolic parameters (insulin sensitivity, insulinemia and cholesterol) and tended to reduce body weight and fat mass [303]. Nonetheless, the investigations on the mechanisms by which these bacteria induce their benefits have mainly focused on the regulation of immune pathways involved in obesity-associated inflammation, generally resulting in metabolic improvements in preclinical trials [304]. Although evidence of the possible action of these bacteria through the modulation of the gut–brain axis is scarce [305,306,307], preclinical studies demonstrated that *A. muciniphila* administration to obese mice increases the intestinal production of the endocannabinoid, 2-arachidonoylglycerol (2-AG); and the endocannabinoid analogue, oleoylglycerol (2-OG) [305]. These ligands can modulate the secretory function of L cells and, theoretically, the activity of vagal afferents [308,309].

Other emerging strategies to identify new probiotics or live bio-therapeutic products tend to mimic drug discovery approaches. In this case, the selection of effector intestinal bacteria is based on in silico predictions (computational molecular modelling) of their capacity to produce bioactive molecules [310]. Based on this strategy, the strain *Hafnia alvei* 4597 was selected to provide the protein ClpB, a bacterial mimetic of α-MSH, which induced satiety. This discovery was based on the initial detection of α-MSH reactive autoantibodies in plasma of humans and rodents [311] and in the in silico screening of the presence of ClpB in members of the family Enterobacteriaceae, including the genus *Hafnia* [312]. Studies in obese mice demonstrated that the administration of *Hafnia alvei* 4597 suppressed food intake and improved the metabolic disturbances associated to obesity [209]. Clinical studies in humans to show the efficacy are underway (clinical trial no. NCT03657186).

In spite of this progress, further advances are still needed to increase the effectiveness and clinical applicability of microbiome-based strategies. In fact, interindividual variability of the human host and its gut microbiota could change the response to these therapies [313]. Therefore, efforts are also underway to develop precision probiotics through a deep understanding of their mode of action and the factors influencing the individual host response [310]. This requires the integration of the person-specific genotypic and phenotypic variables, including microbiome data (strain-level composition, transcriptomics, metabolomics, etc.), that may help to predict the health outcomes of a specific intervention. This strategy may also be applied to the identification of personalized microbiome-based strategies that improve nutrient sensing routes and, thus, control energy homeostasis. To this end, further efforts are needed for the identification of cross-feeding pathways between different intestinal bacteria of a specific host and the resulting dietary and bacterial-derived effector molecules, considering the person’s meal timings and dietary composition.

## Data Availability

Not applicable.
